# Phospholipase-Cγ1 Signaling Protein Down-Regulation by Oligoclonal-VHHs based Immuno-Liposome: A Potent Metastasis Deterrent in HER2 Positive Breast Cancer Cells

**DOI:** 10.22074/cellj.2020.6704

**Published:** 2019-09-08

**Authors:** Ommolbanin Asadpour, Fatemeh Rahbarizadeh

**Affiliations:** 1 Department of Medical Biotechnology, Faculty of Medical Sciences, Tarbiat Modares University, Tehran, Iran; 2 Research and Development Center of Biotechnology, Tarbiat Modares University, Tehran, Iran

**Keywords:** HER2, Liposome, Oligoclonal, Phospholipase Cγ1, VHHs

## Abstract

**Objective:**

The purpose of this study was to develop multivalent antibody constructs via grafting anti-HER2 antibodies, including Herceptin and oligoclonal-variable domain of heavy chain antibodies (VHHs), onto liposome membranes to enhance antibody activity and compare their effect on phospholipase C (PLC) signaling pathway with control.

**Materials and Methods:**

In this experimental study, SKBR3 and BT-474 cell lines as HER2 positive and MCF10A cell line as normal cell were screened with anti-HER2 antibodies, including constructs of multivalent liposomal antibody developed with Herceptin and anti-HER2 oligoclonal-VHHs. To confirm the accuracy of the study, immunofluorescent assay, migration assay and immuno-liposome binding ability to HER2 were evaluated. Finally, the antibodies effect on PLCγ1 protein level was measured by an immunoassay method (ELISA).

**Results:**

In the present study, by using multivalent form of antibodies, we were able to significantly inhibit the PLCγ1 protein level. Interestingly, the results of migration assay, used for study the motility of different types of cell, shows correspondingly decreased number of immigrated cells in SKBR3 and BT-474 cell lines. Since MCF10A cells show no overexpression of HER2, as expected, the result did not show any change in PLCγ1 level. Moreover, immunofluorescent assay has confirmed high expression of HER2 in SKBR3 and BT-474 cell lines and low HER2 expression on MCF10A cell line. High binding of immuno-liposome to SKBR3 and BT-474 cells and low binding to MCF10A confirmed that in this study anti-HER2 antibodies have conserved binding ability to HER2 even after conjugation with liposome.

**Conclusion:**

PLCγ1 protein levels did indeed decrease after treatment with immuno-liposome form of compounds in both two tested cell lines, verifying the inhibition ability of them. Moreover, an elevated antibody activity is associated with liposomes conjugation suggesting that immuno-liposome may be a potential target for enhancing the antibody activity.

## Introduction

Breast cancer is a well-known cancer among women worldwide (1, 2). Amplification of HER2 oncogene, as a member of the epidermal growth factor receptor (EFGR, also knwon as HER) family in human, leads to expansion and progression of the defined offensive types of breast cancer. Moreover, it has been known that HER2 plays a critical role in uncontrolled propagation of cancer cells in breast cancer through dysregulation of HER2-mediated signaling pathways. Hence, in the last decades, HER2 targeting has been applied as a strategy for curing this type of cancer (3). The previous studies showed that HER2 has no specific ligand and activated by homo- or hetero-dimerization with other family members such as HER1 and HER3. In addition, HER2 dimerization results in auto-phosphorylation on tyrosine and cytoplasmic domain residues of the receptors result in a variety of signaling pathways including phospholipase C (PLC) initiates (4, 5). 

PLC, which belongs to membrane-associated enzyme family, plays a remarkable role in signal transduction pathways in response to hormones, growth factors and neurotransmitters. PLC hydrolyzes phospholipid phosphatidylinositol 4 and 5-bisphosphate (PIP2) to produce 1,2-diacylglycerol (DAG) and inositol 1,4,5-trisphosphate (IP3). Therefore, DAG and IP3 act as significant secondary messengers in initiating various cellular processes as well as substrating the synthesis of some important signaling molecules. Based on PLC similarities in their conserved core structure and its different act in the specific domains of each family, they are classified into six isotypes, including PLCß, PLCγ, PLCd, PLCe, PLCγ and PLCγ in mammals (6). The .1 isoform of PLC is one of the popular signaling proteins, with a molecular weight of 145-kDa, encoded by PLCγ1 gene in humans. it is activated in response to growth factors or integrin receptors-dependent pathways (7, 8). Phosphorylation on tyrosine residue 783 of PLCγ1 activates this enzyme to contribute critical roles in cell migration, invasion and spreading in cancers (7, 9, 10). By studying PLCγ1 and the corresponding role in tumors like breast carcinomas, it was clarified that extreme expression of PLCγ1 facilitates cancer metastasis, while blocking this protein will halt the cancer expansion (7, 11). Consequently, PLCγ1 can be considered as a key regulator in cell migration upon RTK signaling and the development of new anti-cancer drugs could be an ongoing research field around this protein (12). 

Recombinant antibody technologies, generating novel drug formats, honored the Nobel Prize in 1970 and considered as a revolution in Immunology (13, 14). A variable domain of heavy chain antibodies (VHH), as the novel member of recombinant antibodies which is found in Camelidae, consists of a single monomeric variable antibody domain, applying selective binding to a specific antigen. Molecular weight of this extraordinary fragment is 15 kDa and it is characterized by 4 nm height and a 2.5 nm diameter and with desirable properties such as convenient cloning, affordable manufacturing, supreme stability and invisible epitopes binding that make it an attractive option in cancer treatment (15-18). 

Monoclonal antibody (mAb) has a monovalent affinity for the same epitope on an antigen which may lead to certain limitations such as resistance and limited efficacy in therapy. In contrast, oligoclonal antibodies, as the new model of this era, mimics the natural immune system and consist of a mixture of mAb clones. Altogether, they show specificity of monoclonal antibodies as well as sensitivity and affinity of polyclonal antibodies. Therefore, a combination of monoclonal antibodies with an oligoclonal-based approach might be more effective than monotherapy (19). 

Multivalent antibody constructs, as a novel product in therapeutic purposes, attracted more attention within the last few years. This structure consists of a suitable surface to bind into 10s-100s of molecules in order to increase the efficiency of the antibody/target complex. When multivalent constructs of antibody subjects tumor antigens, capacity and avidity of the structure culminates due to target/antibody gathering (20). Consequently, they may form the fundamental aspects of developing a cancer therapy in pharmaceuticals. One way to formulate the structure is through the conjugation of antibodies on the surface of a liposome. Liposomes consist of the lipid bilayer membrane surrounding an aqueous core and attaching multiple copies of antibodies on each liposome could provide multi-valency to them (21, 22).

The present study explores an alternative strategy to enhance therapeutic activity of anti-HER2 antibodies, namely combining three distinct VHHs. The next challenge is to develop a multivalent constructs of antibodies that can effectively decrease PLCγ1 protein level compared to the control. 

## Materials and Methods

### Cell lines and culture conditions

In this experimental study, as two HER2-overexpressing breast cancer cell lines, SKBR3 (adenocarcinoma epithelial cells) and BT-474 (ductal carcinoma epithelial cells) were purchased from DMSZ (Braunschweig, Germany). SKBR3 cells were grown in Dulbecco’s Modified Eagle Medium (DMEM, Thermo Fisher Scientific, USA) enriched with 15% fetal bovine serum (FBS, Thermo Fisher Scientific, USA), 10 mg/ml insulin (Sigma- Aldrich, USA) and 1x penicillin-streptomycin (100x solutions, Thermo Fisher Scientific, USA). BT-474 cells were cultured in RPMI-1640 (Thermo Fisher Scientific, USA) supplemented with 10 mg/ml insulin, 20% FBS and 1x penicillin-streptomycin. Moreover, MCF10A (human breast fibrocystic disease/normal epithelial cells) as a HER2-negative model were grown in DMEM/Nutrient Mixture F-12 (DMEM/F12, Thermo Fisher Scientific, USA) completed with 0.001 mg/ml insulin, 20 ng/ml epidermal growth factor (EGF, Peprotech, USA), 5% horse serum (Thermo Fisher Scientific, USA), 500 ng/ml hydrocortisone (Sigma-Aldrich, USA) and 1x penicillin- streptomycin. 

### Purification of anti-HER2 VHHs

The anti-HER2 VHHs clones (RR4, RR3 and RR13) were isolated using phage display technique and transformed in shuffle T7 competent *E. coli* (NEB, USA) (15, 23). Luria-Bertani (LB) broth containing 100 mg/ ml Kanamycin was used to produce a starter culture at 37°C. Then, it was inoculated at a 1:1000 dilution rate into Terrific Broth (TB) contained Kanamycin and incubated at 37°C until optical density (OD)_600 nm _ was reached to 0.5. In the next step, 0.25 mM isopropyl-ß-D-thio-galactoside (IPTG, MW 238g/mol, Sigma-Aldrich, USA) was used to induce protein expression at 18°C overnight. After centrifugation at 5000×g for 15 minutes (4°C), 5 ml lysis buffer (including 50 mM Na_2_HP_4_, 300 mM NaCl, 15 mM imidazole, 1 mM phenylmethane sulfonyl fluoride as a serine protease inhibitor from Sigma-Aldrich, 1% Triton X-100 and 100 mg/ml lysozyme, pH=8.0) was added to each gram of pellet and incubated for 30 minutes at room temperature (RT), followed by sonication (60% power, 2 cycles: 5 minutes with 5 minutes interval on ice). The yield of suspension was centrifuged at 5200×g for 30 minutes (4°C) and the supernatant containing proteins was passed through a 0.45 µm filter immediately before applying to the column. Then, it was applied to pre-equilibrated nickel-nitrilotriacetic acid column (Ni- NTA, Qiagen, Germany) with adsorption buffer (500 mM NaCl, 50 mM NaH_2_PO_4_, 20 mM imidazole, pH=8.0) at 4°C. Next, the column was washed with adsorption buffer (500 mM NaCl, 50 mM NaH_2_PO_4_, 20 mM imidazole, pH=7.5) five column volumes (CV). The adsorbed VHHs were eluted using the imidazole buffer (500 mM NaCl, 50 mM NaH_2_PO_4_, 500 mM imidazole, pH=8.0) two CVs. Purified VHHs were collected with a flow rate of 1 ml/minute, followed by de-saltation and concentration by Amicon filter (EMD Millipore, Germany) using 3 kDa cut-off. Total protein concentration was measured by Bradford assay (24) and finally analyzed by sodium dodecyl sulfate-polyacrylamide gel electrophoresis (SDS-PAGE) (25). Purified VHHs confirmation was approved by western blotting assay (Abcam protocol, UK) using 6x-histidine tag IgG and anti-mouse-HRP antibodies with 3,3'-Diaminobenzidine (DAB, Sigma-Aldrich, USA). 

### Liposome preparation and characterization

Liposomes were composed of dipalmitoylphosphatidylcholine (DPPC), DSPE-PEG (2000) maleimide, cholesterol, 1.2-distearoyl-snglycero- 3-phosphoethanolamine-N-(amino(polyethylene glycol)-2000) (DSPE-PEG2000) with respectively 7, 0.1, 2.5 and 0.4 µmol volume, obtaining from Avanti Polar Lipids (USA). After dissolving in chloroform and methanol solutions (rate of 9:1 v/v, both from Sigma- Aldrich, USA), thin biofilm was formed in a round-bottom flask. After evaporation of the resulting suspension, a rotary evaporator under low pressure (45°C, 70 rpm) was used up to completely removing the solvents. In continue, the produced biofilm was hydrated in 1.2 ml sodium phosphate buffer (including 50 mM NaH_2_PO_4_, 0.15 mM NaCl and 1 mM EDTA, pH=7.0) at 70°C resulting in spontaneously organized multi-lamellar vesicles (MLVs). Finally, the MLVs were extruded 21 times at 65°C through 0.1 µm pore sized polycarbonate membranes (Avanti Polar Lipids, USA) using an Avanti’s mini-extruder (Avanti Polar Lipids) to form small uni-lamellar vesicles. After incubation of the liposomes at RT to cool-down, they were stored at 4°C. Produced liposome diameters were defined by a Zetasizer Nano APS (Malvern Instruments Ltd, UK) at 25°C following the appropriate dilution with phosphate buffered saline (PBS). 

### Synthesis of immuno-liposomes

Anti-HER2 oligoclonal-VHHs and Herceptin (a mAb against HER2) were thiolated using 2-iminothiolane hydrochloride (Traut’s reagent, Sigma-Aldrich, USA) in sodium borate buffer (composed of 0.15 M H_3_BO_3_ and 1 mM EDTA, pH=8.3) by incubating for 60 minutes at RT. The buffer was next concentrated and exchanged withsodium phosphatase buffer (including 50 mM NaH_2_PO_4_, 0.15 mM NaCl, 1 mM EDTA, pH=7.0) using appropriateAmicon filters (EMD Millipore, USA) with respectively3 and 100 kDa cut-off. Thiolated antibodies were used in conjugation with liposomes at a molar ratio of 10:1(2-iminothiolane: antibody). In order to do this, 50 mg of theprepared liposomes containing maleimide-terminated linkerwas mixed with 1.7 mg/ml of thiolated VHHs and 1 mg/mlof thiolated Herceptin under constant gentle shaking for 1hour at RT, following unconjugated antibodies eliminationby ultra-centrifugation at 30000×g for 1 hour. The samplevolumes were adjusted to 1 ml with the mentioned sodiumphosphate buffer and PEGylated immuno-liposomes were sterilized by transmission through a 0.22 mm sterile filter and stored at 4°C. In continue, SDS-PAGE following on silver staining was used for confirmation of conjugation(26). The zeta potential and average size of PEGylated immuno-liposome were calculated using a dynamic light scattering technique (DLS) at maximum 830 nm laser sources and a scattering angle of 90° at RT. Three different tests were done for each estimation. The amount of bounded antibodies to liposome was calculated as described by Allen et al. (27) considering that the diameter of 17 kDa VHH molecule was around 14.2 A° and sum of the area of a cholesterol molecule and phospholipid in liposome was 81 A° for a DPPC:cholesterol, in 1:1 molar ratio (the area of polar head for phospholipid and cholesterol were respectively 72 A° and around 19 A°). 

### *In vitro* fluorescent imaging of liposomes 

In order to determine binding ability of anti-HER2 antibodies, the liposomes were labeled by PKH67 green fluorescent cell linker kit (Sigma-Aldrich, USA) as described in the manufacturer’s handbook with some modifications. Briefly, 1 mg liposomes was washed twice in PBS and centrifuged at 400×g for 5 minutes to obtain a loose pellet. The supernatant was then carefully aspirated and liposome was suspended in 1 ml diluent C staining vehicle (included in the kit; it is a solution to maximize dye solubility and efficiency) via gentle pipetting. After preparing 2x Dye solution (4×10^–6^ M in diluent C) immediately and before staining, liposome suspension was mixed with the dye solution (1:1) and incubated for 5 minutes with periodic mixing. The staining was stopped by adding an equal volume of 1% bovine serum albomine (BSA) for one minute. Then, the suspension was centrifuged at 400×g for 10 minutes (RT) in order to omit excess dies. Finally, the supernatant was removed and washed liposome was suspended in 10 ml PBS. 48 hours prior to preparing fluorescent-labeled liposome, SKBR3 and MCF10A cells were cultured in a 24-well plate (10000 cells per well). The growth medium was replaced with fresh medium containing 500 µg/ml of each labeled non-conjugated liposome, labeled Herceptin-conjugated liposome and labeled oligoclonal-VHHs-conjugated liposome. After 15 minutes, the cells were rinsed with PBS (pH=7.4) once and visualized using fluorescence microscopy at 635 nm wavelength. 

### Immunofluorscent studies of the fixed cultured SK-BR-3, BT-474 and MCF10A cells 

Immunofluorscent protocol (Abcam, UK) was used to quantitate HER2 amplification on SKBR3, BT-474 and MCF10A cell lines. Briefly, the cells were grown on chambered cell culture slides (Green Bio Research, USA) to a density of 10000 cells/cm^2^ in the aforesaid medium. The monolayer cells were fixed in 4% paraformaldehyde (PFA, Merck, Germany) in PBS (pH=7.3) for 20 minutes on ice. The fixed monolayer cells were blocked and permeabilized by incubation in 3% BSA, 0.1% Triton X-100 in PBS (pH=7.3) at RT for 20 minutes. Then, the cells were reacted with rabbit Anti-ErbB2 mAb (EP1045Y; final concentration of 1:250) primary antibodies (Abcam, UK) for 60 minutes at RT. Finally, 4',6-diamidino-2phenylindole (DAPI, 1:10000, Sigma-Aldrich, USA) was added and incubated for five minutes. The fluorescence was detected by fluorescence microscopy at 635 nm wavelength. 

### Cell migration assay

Cell migration assay was done in transwell polycarbonatemembrane inserts (tissue-culture-treated, 24 well format, 8 µmpores, Sigma-Aldrich, USA) coated with 40 µg/ml collagenI (Sigma-Aldrich, USA). SKBR3 and BT-474 cells werepretreated with the mentioned amount of different treatmentsfor 30 minutes and they were subsequently detached. The cellswere then suspended in DMEM and RPMI-1640 containingthe treatments, added (20000 cells/100 µl) to the top of eachmigration chamber, and allowed to migrate. After 2 and 24hours, the membrane drained out. The cells, which had not migrated, were removed with a cotton swab. The cells on theinsert filter were fixed with 4% paraformaldehyde, stained with 1% crystal violet and then counted. 

### Total PLCγ1 protein expression assay

To determine total PLCγ1 protein content by westernblotting (Abcam, UK), the membranes were blotted with antiPLCγ1 mouse primary mAb (Merk, Germany) visualizedwith anti-rabbit horseradish peroxidase (HRP)-conjugatedgoat secondary antibody (Elabscience, China). The proteinbands were detected using the enhanced chemiluminescence(ECL) western detection system (Amersham PharmaciaBiotech, USA). .-actin antibody (Cell Signaling Technology, USA) was used as housekeeping protein.

### PLCγ1 protein immunoassay 

Antigen binding ability of the prepared immuno-liposomeswas immediately studied after labeling. 500000 cells fromeach BT-474, SKBR3 and MCF10A line were seeded in T25 flasks and incubated for 24 hours. Then, the cells were subjected to 7.37 µg/ml, 19.61 µg/ml, 26.12 µg/ml, 10µg/ml and 38.7 µg/ml of respectively RR3, RR4, RR13, Herceptin and oligoclonal-VHHs final concentration. Inaddition, 50 mg/ml of non-conjugated liposome, Herceptinconjugated liposome and oligoclonal-VHHs-conjugatedliposome were added to each flask. Herceptin concentrationwas chosen based upon the previously reported data (28)þ, and VHH concentrations were based upon the mass ratiobetween Herceptin (160 kDa) and VHHs (RR3, 16.9 kDa; RR4, 15.7 kDa and RR13, 16.9 kDa) and the cells wereapproximately at the same viability and confluence on theday of treatment. After 2 and 24 hours, the medium wasdiscarded and the cells were washed with PBS (pH=7.4). Then, the washed cells were lysed with RIPA Buffer 10X(Cell Signaling Technology). Total protein concentration wasdetermined by Bradford method. To detect PLCγ1 protein anenzyme-linked immunosorbent assay (ELISA) kit was usedfor human specific PLC gamma 1 (PLCG1, Cloud-CloneCorp., USA) relying on a sandwich enzyme immunoassayfor *in vitro* quantitative measurement of PLCγ1 in somebiological fluids. The procedure was done according tothe kit handbook (SEA269hu 96 Tests). The standardsor samples were added to microtiter the plate containingbiotin-conjugated PLCγ1antibody. Then, HRP conjugatedavidin solutions were added to each well and incubated for 30 minutes at 37°C. Only the color of those wells thatcontained PLCγ1 protein was changed. The reaction wasstopped using 0.2 M sulphuric acid. Finally, the level of color changing was measured spectrophotometrically at OD_450 nm_. The concentrations of PLCγ1 in the samples were determined comparing to the used standard curve.

### Statistical analysis

Statistical analysis was carried out using SPSS for windows, version 16.0 (SPSS Inc., USA). A one-way ANOVA, followed by the least significant difference (LSD) test was used to compare different groups. Levels of P<0.05, P<0.01 and P<0.001 were considered statistically significant. Data are expressed as mean ± SD. 

## Results

### Anti-HER2 VHHs expression and purification

**Fig.1:**
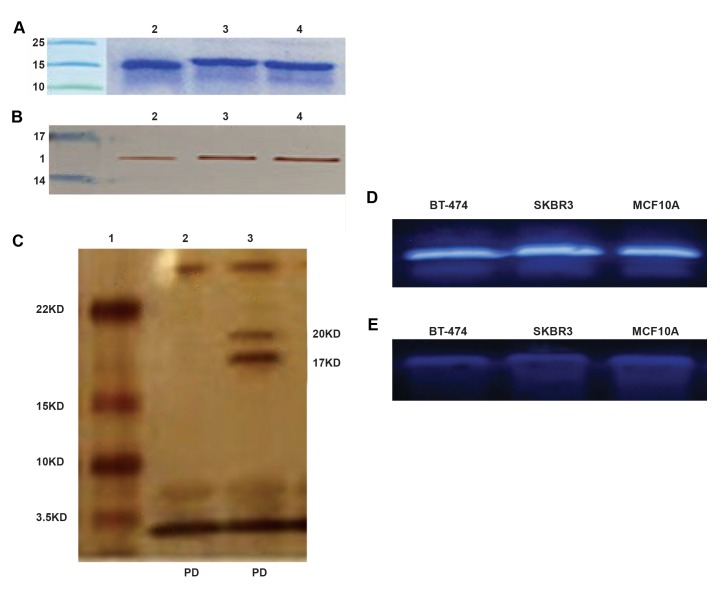
Expression and purification of VHHs and confirmation of its conjugation to liposome. A. SDS-PAGE analysis of anti-HER2 VHHs purification using nickel affinity chromatography. Lane 1; Molecular weight of protein markers, Lane 2; RR3, Lane 3; RR4, and Lane 4; RR 13, B. Western blotting analysis of anti-HER2 VHHs using 6x-histidine tag IgG and anti-mouse-HRP antibodies with DAB. Lane 1; Protein molecular weight marker, Lane 2; RR3, Lane 3; RR4, Lane 4; RR13, C. Confirmation of anti-HER2 VHHs conjugation on the surface of liposome by SDS-PAGE silver staining. Lane 1; Protein molecular weight marker, Lane 2; Non-conjugated liposome, Lane 3; VHHsconjugated liposome and phospholipid debris, D. Detection of B-actin, and E. Total PLCγ1 expression in different breast cancer cell lines by western blotting. SDS-PAGE; Sodium dodecyl sulphatepolyacrylamide gel electrophoresis and VHH; Variable domain of heavy chain antibodies.

A single protein band was obtained for each VHH with theexpected molecular mass, as shown in the figure obtainedfrom 12% SDS-PAGE (Fig .1A) using Coomassie bluestaining. The Bradford assay results indicated obtainingalmost 2.13 mg/ml, 1.95 mg/ml and 1.35 mg/ml protein forrespectively RR3, RR4 and RR13 per 250 ml of bacterialculture. The validity of VHHs purification was confirmed byImmunoblot detection using mouse anti-6x his-tag IgG andanti-mouse-HRP antibodies, showing the bands around 17kDa. This confirms successful expression and purification of the soluble VHHs (Fig .1B-E).

### Characterization of liposomes 

#### Zeta potential and particle size 

**Fig.2:**
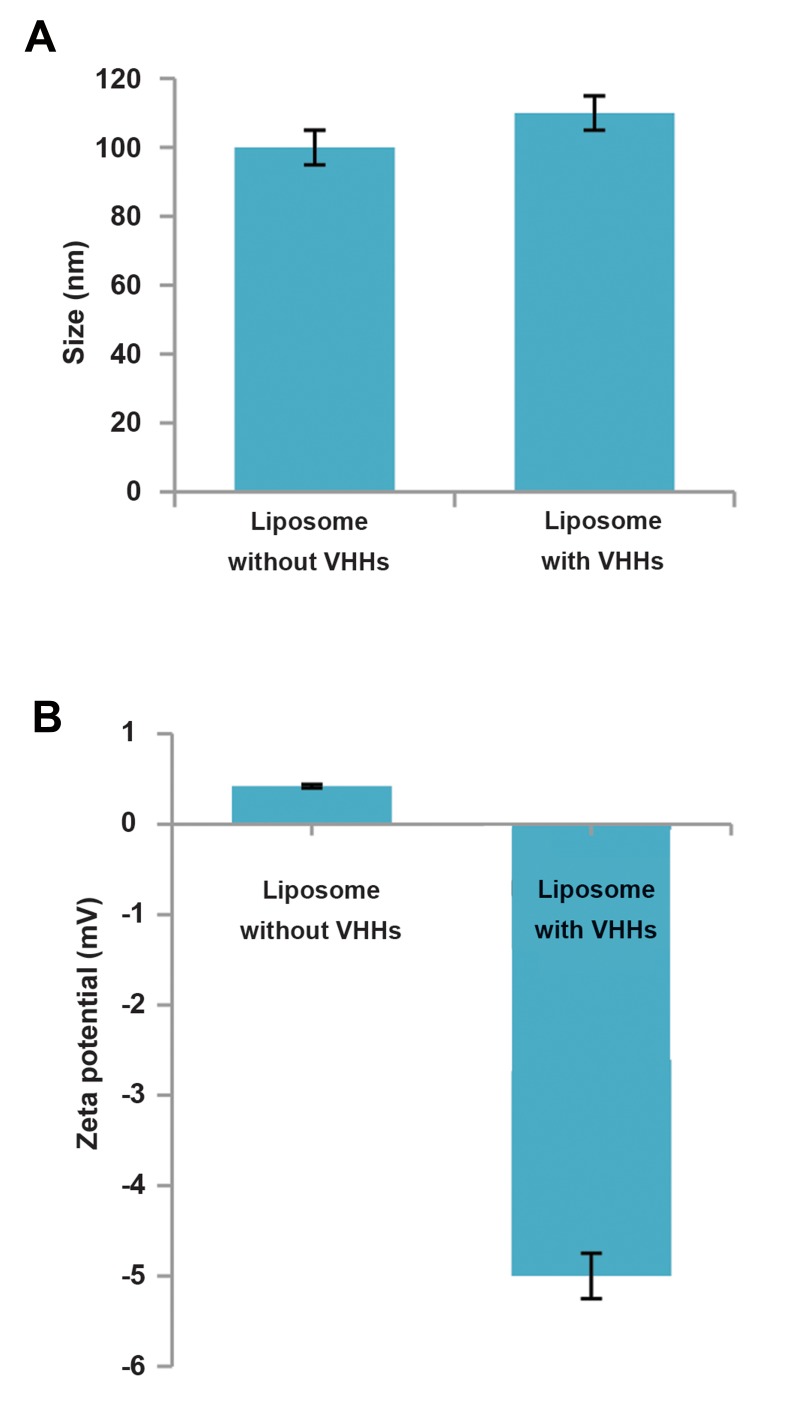
Physicochemical characterization of PEGylated liposome and PEGylated immuno-liposome. **A.** Mean size and **B.** Zeta potential. Data are expressed as the mean ± SD (n=3).

Zeta potential and particle size, as two most important characterization parameters, help predict the stability and act of liposomes. Both of the size and zeta potential results showed excellent reproducibility after three times repetition (Fig .2). The results indicated a monodisperse system for the naked PEG derived liposome with the size below 100 ± 10 nm, corresponding to the diameter of polycarbonate filter, but the particle size of antibody conjugated liposome was in a mean size of 110 ± 10 nm. This confirmed VHHs conjugation with PEGylated liposomes.

Zeta potential measures the protein electrophoretic mobility that is defined by the overall charge of a particle in a particular medium. So, any subsequent modification of the liposome surface can be monitored through its measurement. While working with the cell lines, it is preferable to have a ZP which should not be too much negative, since the cell membrane is already negatively charged; thus, it causes more interactions between nano-carrier and the cell. In this study, the mean zeta potential of the naked PEG derived liposome was 0.42 ± 0.1 mV which is close to neutral range. However, it was decreased into -5 mv after liposomes modification by VHHs indicating that the VHHs induce a negative charge on the surface of liposomes to minimize nonspecific interaction with cell membrane. 

### Determination of oligoclonal-VHHs liposome conjugation

Integrity of VHHs on liposome was confirmed by SDSPAGE, followed by silver staining (Fig .1). The conjugated VHHs molecular weight of the band was around 20 kDa which was larger than free VHHs molecular weight (16.9 kDa). VHHs molecular weight was approximately increased 3kDa, due to the ligation of VHHs to MalPEG2000- DSPE. This finding showed that intact form of the VHHs was efficiently incorporated into the liposome. 

### *In vitro* fluorescent imaging of liposomes

**Fig.3:**
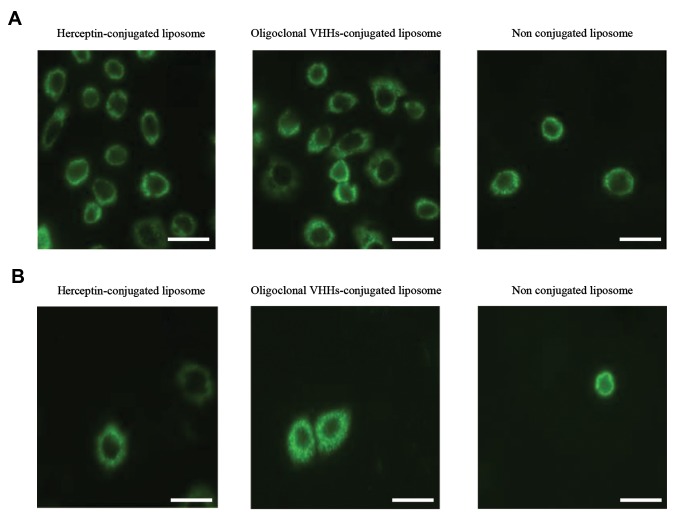
Representation of binding ability of PKH67 labeled Herceptin-conjugated liposome, oligoclonal VHHs-conjugated liposome and non-conjugated liposome on HER2-positive and HER2-negative cells using Nikon EcliPSE Ti fluorescence microscopy (Nikon, Japan), on A. SKBR3 and B. MCF10A cells (scale bar: 0.1 μm).

Fluorescence-labeled liposomes were prepared from a homogeneous population of uni-lamellar liposomes by incorporating PKH67 green fluorescent dye into the liposomal phospholipid bilayer. By using a fluorescence microscopy, it was shown that cultured cells were labeled with fluorescent liposomes and a clear shine was observed (Fig .3). The results indicated strong observation of fluorescence in SKBR3 cells, despite MCF10A cells confirmed specific binding ability of immuno-liposomes. Moreover, obtaining the similar fluorescence by Herceptin and VHHs showed comparable HER2 binding ability of VHHs against Herceptin. 

### Immunofluorescent analysis of the fixed cultured cells

**Fig.4:**
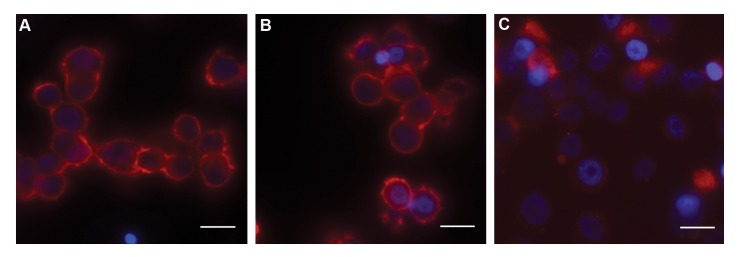
Immuno-fluorescent analyses of HER2 production using fluorescent anti-ErbB2 antibody [EP1045Y] ,on the fixed cultured cells of A. SK-BR-3, B. BT474, and C. MCF-7 cell lines by Nikon EcliPSE Ti fluorescence microscopy. The results of this test shows high-expression of HER2 on the surface of SKBR3 cells and BT-474 cells, cells, and Very low-expression for MCF10A cells.

In the preparation of the fixed cells, Immunofluorescent experiments with anti-ErbB2 antibody [EP1045Y] detected high levels of HER2 protein in SKBR3 and BT-474 as HER2 positive cell lines, compared to the MCF10A as a normal cell line (Fig .4). A notably high- strength of HER2 fluorescence signal were localized to the cell membrane in SKBR3 and BT-474 cell lines, whereas only low signal levels were found in the MCF10A control. 

### Immuno-liposomes detracted cell migration

**Fig.5:**
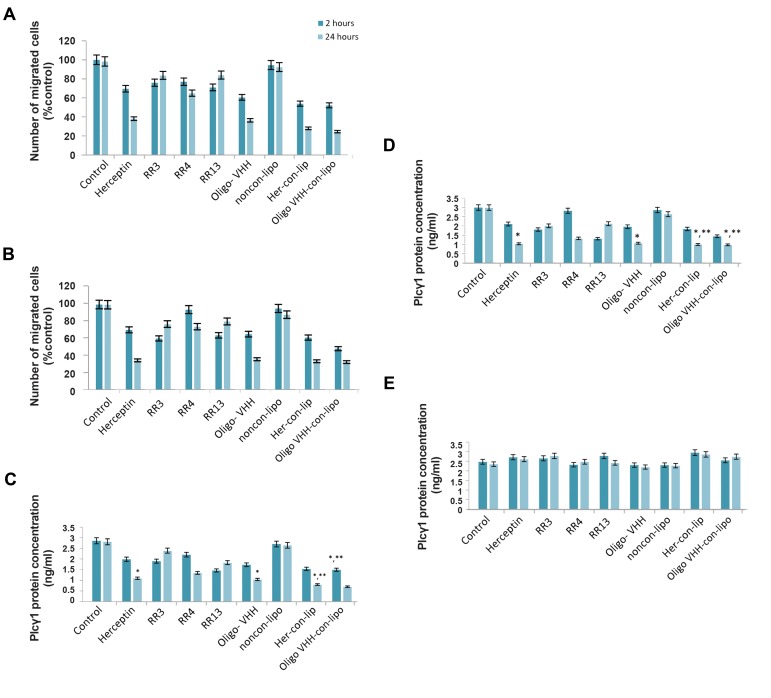
Functional characterization of immunoliposomes. A. Cell migration assay in BT-474 cells, B. Cell migration assay in SKBR3 cells. *In vitro* treatment of cancer cells with monovalent or liposomal antibody constructs including Herceptin, RR3, RR4, RR13, oligoclonal-VHHs (oligo- VHH), non-conjugated liposome (non-con-lipo), Herceptin conjugated liposome (Her-con-lip) and oligoclonal-VHHs conjugated liposome (oligo VHH-con-lip) in C. BT-474, D. SKBR3, as HER2 positive cell lines, and E. MCF10A, as a normal cell line. The total cell numbers, migrated cells and plc.1 protein concentration were quantitated after 2 and 24 hours of different treatments. *; P<0.05 and **; P<0.01 shows the significant decrease after treatment by Herceptin and oligoclonal-VHHs individually or in conjugation with liposome against control. Data show mean ± SD.

We next tested the effect of different anti-HER2 antibodies on cell migration in SKBR3 and BT-474 cell lines. Treatment with Immuno-liposomes specifically reduced the cell migration as well as Herceptin and oligoclonal-VHHs in both cell lines (Fig .5). No inhibition was observed when the other treatments were used in comparison with the control. 

### Total PLCγ1 protein expression

High levels of PLCγ1 protein expression were detected in the SKBR3 and BT-474 positive cells for HER2 whereas the corresponding bands in the MCF10A control cells were very low (Fig .1E). All cell lines expressed clear and distinct bands of B-actin (Fig .1D) indicating integrity of the assay. 

### PLCγ1 protein immunoassay

The effects of free and liposome conjugated anti-HER2 antibodies on PLC pathway were studied in SKBR3, BT-474 and MCF10A cells via PLCγ1 protein level, as PLC pathway downstream target introduced as an essential factor for metastasis development and cancer progression (6). As the results showed (Fig .5), in both of HER2 positive cells, oligoclonal-VHHs and Herceptin individually or in liposome conjugated form, decreased PLCγ1 protein level in comparison with the untreated cells (SKBR3: Herceptin P=0.028, oligoconal-VHHs P=0.031, Herceptin conjugated liposome P=0.026 and oligoclonal-VHHs conjugated liposome P=0.025, BT-474: Herceptin P=0.04, oligoconal-VHHs P=0.034, Herceptin conjugated liposome P=0.011, and oligoclonal- VHHs conjugated liposome P=0.018). The result did not show any change in PLCγ1 level of MCF10A cells, since these cells showed no overexpression of HER2. In addition, activity of immuno-liposomes was magnified. In this case, the liposome form which antibodies were cumulated on its surface, increased the effect of the antibodies. 

The charts represent results of different treatments in the three indicated cell lines (Fig .5). This observation collaborates with the fact that free antibodies induced a decline in the quantity of PLCγ1 protein in HER2 positive cells, emphasizing that liposomal conjugated antibodies decreased it even more significantly. It is worthy to say that differences in the ability of individual antibody and immuno-liposomes in PLC cell signaling pathway modulation could be therapeutically important (29, 30). 

## Discussion

Previous studies suggested that overexpression of PLCγ1 protein is one of the key factors in cellular migration and invasion. It can be proposed as a vital enzyme in the development and maintenance of tumor metastasis (11). Despite it has been shown that PLCγ1 is activated by HER2 (1), in practice, no dedicated HER2 target has been introduced to control the activity of PLCγ1 protein (3) and it seems urgent to consider this issue. 

The present study has investigated the effect of multi- capacity immuno-suppressive agents carrying anti-HER2 compounds *in vitro*. As observed, oligoclonal-VHHs and Herceptin can decrease the level of the PLCγ1 protein and immuno-liposomal application also intensifies this effect. Finding similar results in BT-474 and SKBR3 cancer cells can confirm the ability to strengthen antibody- based therapies using immuno-liposomal technology. One of the most important advantages of the multiplicity structure antibody is the increased binding reliability compared to single antibodies (31). Although clinical trials of mAb therapy have provided the best hope for increasing the clinical benefits of antibodies, especially for inhibiting signaling via tyrosine kinase receptors (32), enhancing their performance in some features such as stability, affinity, specificity and size as well as their pharmacokinetic properties are still being studied and the demand for introducing suitable alternatives has become a challenge. In this case, discovery of heavy chain antibodies in camel species has created a new opportunity (33). In comparison with conventional antibodies, heavy chain antibodies have been completely evolved in the absence of light chains, while their unique biophysical and pharmacologic properties have categorized these molecules as a new member of antibody-based therapy agents enabling them to gradually make obsolute commonly used therapeutic antibodies (34). Additionally, improvement in the expression and purification of oligoclonal antibody mixtures in the field of therapeutic agent productions provides an opportunity for imitation of the natural immune system and oligoclonal VHHs are suggested as a good tool for improving overall response. Recently, a clinical trial combined Pertuzumab and Trastuzumab with high affinity against different subdomains of the HER2 extracellular domain and reported a 24.2% response rate in HER2-positive breast cancer patients (15). Moreover, other clinical trial showed that non-overlapping binding of two anti-EGFR monoclonal antibodies promoted reduction of receptor expression on the cell surface. Furthermore, combination of Pertuzumab and Trastuzumab oligoclonal antibodies blocked HER2-dependent signals much more efficiently compared to its individual components (35). 

In recent years, several strategies have been established to improve the efficiency of antibody-based therapies, among which immuno-liposomes, as a strong approach, have potential to produce multi-dose antibodies to enhance the action of antibody-based therapies. They have also been appeared as a common thread for a broad range of biological processes which can mediate the multiplicative interactions of cellular signaling by developing the cross-linking of antibody/target complex (6). Chiu et al. (21) examined the potency of free and liposomal form of Trastuzumab to detract the expression levels of HER2 and Akt, as respectively a target and downstream molecule. She demonstrated that multiplicity of liposomal Trastuzumab can reduce active regulation of the Akt phosphorylated form. 

One of the remarkable cases in this study is the observation of different behavior of individual VHHs in HER2-positive cells. As the results show, RR3 and RR13 showed a decrease after two hours, followed by slightly increase in the amount of PLCγ1 protein level after 24 hours. This could strongly suggest different epitopes diagnosis and their effect on PLCγ1 protein in these two VHHs, while the effect of oligoclonal VHHs showed an increase in 2 and 24 hours. The challenge of differences in their behavior might be answered by considering the overall synergistic effect of oligoclonal-VHHs. 

Prior to investigating the therapeutic effect of immuno-liposomes, it was necessary to clarify some characteristics of experiment material, including: i. The ability of immuno-liposome binding after conjugation with antibodies. Since using fluorescence microscope is considered as an effective tool for assessing the cumulative effect of liposomes (36), preparation of the labeled immuno-liposomes was performed using a PKH67 fluorescence label, which specifically affects the lipid profile of membrane, and the liposome lipid structure allows application of this fluorescent (37). According to the results, observing stronger fluorescence in SKBR3 compared to MCF10A cells confirmed successful attachment of the antibodies to the liposomal surface (8, 38). ii. Immunofluorescent method was used to study HER2 protein level on two breast carcinoma cell lines: SK-BR-3 and BT-474 compared to MCF10A, as a normal cell line. The first two cell lines were characterized in terms of higher HER2 protein content, whereas MCF10A cells have a very low HER2 protein content. The method presented here compares high and low protein content by analyzing relative intensity of signals. 

iii. One of the considerable experiments was to determine total PLCγ1 protein in cell lysates by western blotting method, when an internal calibrator is included in the assay systems. .-actin antibody showed a band with the same intensity in different treatments providing accurateness in the sample quantification among different assays. Using this antibody provides a good reference for confirming the correctness of total PLCγ1 protein level estimation in different samples. By comparing the results of total and phosphorylated form of PLCγ1 protein level, it is suggested that the inhibitory effect of this protein might not be due to the inhibition of total PLCγ1 synthesis, but it happens after the protein phosphorylation.

iv. In terms of cell migration, some studies showed a relation between PLCγ1 protein and cell migration indicating the importance of considering immigration ability, in presence of HER2.

Therefore, considering the above indications, immunoliposome has been proposed as a construct of multi- capacity antibody and it can be considered as a demanding intermediary in signaling pathways associated with cell metastasis. 

## Conclusion

These observations are along with previous studies and prepare a stimulating prospect for improving the avidity of antibodies by enhancing the quantity of binding of antibodies to antigens, especially in oligoclonal form, in comparison with single antibodies. Oligoclonal-VHHsconjugated liposome showed a significant elevated affinity in comparison with oligoclonal-VHHs itself. It indicates the effectiveness of these nanoparticles in targeting the HER2 receptor. Furthermore, the observation of similar results between conjugated liposomes with oligoconal- VHHs and conjugated liposomes with Herceptin suggests the possibility of comparative effects of these two antibodies on HER2 positive cancer cells. Based on the results, this study might lead to the expansion of a clinically relevant nanomaterial, whereby PLCγ1, as an effective metastasis factor, is a suitable candidate for targeting. In addition, similar effect of oligoclonal-VHHs and Herceptin in liposome conjugation form remarkably brings new hopes to treat breast cancer with higher efficiency potential by using this approach. 

## References

[1] Ferlay J, Soerjomataram I, Dikshit R, Eser S, Mathers C, Rebelo M (2015). Cancer incidence and mortality worldwide: sources, methods and major patterns in GLOBOCAN 2012. Int J Cancer.

[2] Salouti M (2017). Radioimmunoscintigraphy of breast cancer. J Immun Serum Biol.

[3] Mitri Z, Constantine T, O’Regan R (2012). The HER2 receptor in breast cancer: pathophysiology, clinical use, and new advances in therapy. Chemother Res Pract.

[4] Li SG, Li L (2013). Targeted therapy in HER2positive breast cancer. Biomed Rep.

[5] Tai W, Mahato R, Cheng K (2010). The role of HER2 in cancer therapy and targeted drug delivery. J Control Release.

[6] Falkenburger BH, Jensen JB, Dickson EJ, Suh BC, Hille B (2010). Symposium review: phosphoinositides: lipid regulators of membrane proteins. J Physiol.

[7] Burgess WH, Dionne CA, Kaplow J, Mudd R, Friesel R, Zilberstein A (1990). Characterization and cDNA cloning of phospholipase C-gamma, a major substrate for heparin-binding growth factor 1 (acidic fibroblast growth factor)-activated tyrosine kinase. Mol Cell Biol.

[8] Bristol A, Hall SM, Kriz RW, Stahl ML, Fan YS, Byers MG (1988). Phospholipase C-148: chromosomal location and deletion mapping of functional domains.Cold Spring Harb Symp Quant Biol.

[9] Seidman A, Hudis C, Pierri MK, Shak S, Paton V, Ashby M (2002). Cardiac dysfunction in the trastuzumab clinical trials experience. J Clin Oncol.

[10] Walker K, Boyd NH, Anderson JC, Willey CD, Hjelmeland AB (2018). Kinomic profiling of glioblastoma cells reveals PLCG1 as a target in restricted glucose. Biomark Res.

[11] Sala G, Dituri F, Raimondi C, Previdi S, Maffucci T, Mazzoletti M (2008). Phospholipase Cgamma1 is required for metastasis development and progression. Cancer Res.

[12] Kang DS, Yang YR, Lee C, Park B, Park KI, Seo JK (2018). Netrin‐1/ DCC‐mediated PLCγ1 activation is required for axon guidance and brain structure development. EMBO Rep.

[13] Koch J, Tesar M (2017). Recombinant antibodies to arm cytotoxic lymphocytes in cancer immunotherapy. Transfus Med Hemother.

[14] Cha SW, Bonissone S, Na S, Pevzner PA, Bafna V (2017). The antibody repertoire of colorectal cancer. Mol Cell Proteomics.

[15] Jamnani FR, Rahbarizadeh F, Shokrgozar MA, Ahmadvand D, Mahboudi F, Sharifzadeh Z (2012). Targeting high affinity and epitope-distinct oligoclonal nanobodies to HER2 over-expressing tumor cells. Exp Cell Res.

[16] Heidari Z, Salouti M (2012). Targeting molecular imaging of breast cancer by radioimmunodetection method in nuclear medicine. Current Molecular Imaging.

[17] Van Audenhove I, Gettemans J (2016). Nanobodies as versatile tools to understand, diagnose, visualize and treat cancer. EBioMedicine.

[18] Bannas P, Hambach J, Koch-Nolte F (2017). Nanobodies and nanobodybased human heavy chain antibodies as antitumor therapeutics. Front Immunol.

[19] Gupta RK, Glassy MC, Dübel S, Reichert JM (2014). Oligoclonal and polyclonal antibody preparations. Handbook of therapeutic antibodies.Germany: Wiley-VCH Verlag GmbH & Co.KGaA.

[20] Saeed M, van Brakel M, Zalba S, Schooten E, Rens JA, Koning GA (2016). Targeting melanoma with immunoliposomes coupled to anti-MAGE A1 TCR-like single-chain antibody. Int J Nanomedicine.

[21] Chiu GN, Edwards LA, Kapanen AI, Malinen MM, Dragowska WH, Warburton C (2007). Modulation of cancer cell survival pathways using multivalent liposomal therapeutic antibody constructs. Mol Cancer Ther.

[22] Kontermann R (2006). Immunoliposomes for cancer therapy. Curr Opin Mol Ther.

[23] Nikkhoi SK, Rahbarizadeh F, Ahmadvand D (2017). Oligo-clonal nanobodies as an innovative targeting agent for cancer therapy: new biology and novel targeting systems. Protein Expr Purif.

[24] Bradford MM (1976). A rapid and sensitive method for the quantitation of microgram quantities of protein utilizing the principle of protein-dye binding. Anal Biochem.

[25] Laemmli UK (1970). Cleavage of structural proteins during the assembly of the head of bacteriophage T4. Nature.

[26] Chevallet M, Luche S, Rabilloud T (2006). Silver staining of proteins in polyacrylamide gels. Nat Protoc.

[27] Allen LG, Hovey T, Love MS, Smith JTW (1995). The life history of the spotted sand bass (Paralabrax maculatofasciatus) within the Southern California Bight. CalCOFI Rep.

[28] Yakes FM, Chinratanalab W, Ritter CA, King W, Seelig S, Arteaga CL (2002). Herceptin-induced inhibition of phosphatidylinositol-3 kinase and Akt Is required for antibody-mediated effects on p27, cyclin D1, and antitumor action. Cancer Res.

[29] Wilson CH, Ali ES, Scrimgeour N, Martin AM, Hua J, Tallis GA (2015). Steatosis inhibits liver cell store-operated Ca2+ entry and reduces ER Ca2+ through a protein kinase C-dependent mechanism. Biochem J.

[30] Burgess WH, Dionne CA, Kaplow J, Mudd R, Friesel R, Zilberstein A (1990). Characterization and cDNA cloning of phospholipase C-gamma, a major substrate for heparin-binding growth factor 1 (acidic fibroblast growth factor)-activated tyrosine kinase. Mol Cell Biol.

[31] Niwa T, Kasuya Y, Suzuki Y, Ichikawa K, Yoshida H, Kurimoto A (2018). Novel immunoliposome technology for enhancing the activity of the agonistic antibody against the tumor necrosis factor receptor superfamily. Mol Pharm.

[32] Shuptrine CW, Surana R, Weiner LM (2012). Monoclonal antibodies for the treatment of cancer. Semin Cancer Biol.

[33] Cortez-Retamozo V, Backmann N, Senter PD, Wernery U, De Baetselier P, Muyldermans S (2004). Efficient cancer therapy with a nanobody-based conjugate. Cancer Res.

[34] Moghimi SM, Rahbarizadeh F, Ahmadvand D, Parhamifar L (2013). Heavy chain only antibodies: a new paradigm in personalized HER2+ breast cancer therapy. Bioimpacts.

[35] D’souza JW, Robinson MK (2015). Oligoclonal antibodies to target the ErbB family. Expert Opin Biol Ther.

[36] Kamaly N, Kalber T, Ahmad A, Oliver MH, So P-W, Herlihy AH (2008). Bimodal paramagnetic and fluorescent liposomes for cellular and tumor magnetic resonance imaging. Bioconjug Chem.

[37] Dean-Colomb W, Esteva FJ (2008). Her2-positive breast cancer: herceptin and beyond. Eur J Cancer.

[38] Shi C, Gao F, Gao X, Liu Y (2015). A novel anti-VEGF165 monoclonal antibody-conjugated liposomal nanocarrier system: Physical characterization and cellular uptake evaluation in vitro and in vivo. Biomed Pharmacother.

